# Thermal-solutal-induced bistability of evaporating multicomponent liquid thin films

**DOI:** 10.1073/pnas.2418487122

**Published:** 2025-02-07

**Authors:** Yuki Wakata, Feng Wang, Chao Sun, Detlef Lohse

**Affiliations:** ^a^New Cornerstone Science Laboratory, Center for Combustion Energy, Key Laboratory for Thermal Science and Power Engineering of Ministry of Education, Department of Energy and Power Engineering, Tsinghua University, Beijing 100084, China; ^b^Department of Engineering Mechanics, School of Aerospace Engineering, Tsinghua University, Beijing 100084, China; ^c^Department of Physics of Fluids, Max Planck–Center for Complex Fluid Dynamics, J.M. Burgers Centre for Fluid Dynamics, Faculty of Science and Engineering, University of Twente, Enschede 7500AE, The Netherlands; ^d^Max Planck Institute for Dynamics and Self-Organization, Göttingen 37077, Germany

**Keywords:** multicomponent liquid, evaporating liquid film, Marangoni effect, Bénard–Marangoni convection

## Abstract

Volatile multicomponent liquid films show rich dynamics, due to the complex interplay of gradients in temperature and in solute concentrations. Here, we study the evaporation dynamics of a tricomponent liquid film, consisting of water, ethanol, and trans-anethole oil (known as “ouzo”). With the preferential evaporation of ethanol, cellular convective structures are observed both in the thermal patterns and in the nucleated oil droplet patterns. However, the feature sizes of these two patterns can differ, indicating dual instability mechanisms dominated by either temperature or solute concentration. Using numerical simulations, we quantitatively compare the contributions of temperature and solute concentration on the surface tension. Our results reveal that the thermal Marangoni effect predominates at the initial evaporation stage, resulting in cellular patterns in thermal images, while the solutal Marangoni effect gradually becomes dominant. By regulating the transition time of this thermal-solutal-induced bistability and the nucleation time of oil microdroplets in the ternary mixture, the oil droplet patterns can be well controlled. This capability not only enhances our understanding of the evaporation dynamics but also paves the way for precise manipulation of nucleation and deposition processes at larger scales.

## Significance

The evaporation of liquid thin films is a critical process in various industrial and scientific applications, including coating technologies, microelectronics, and thermal management systems. Due to possible temperature and concentration gradients, the Marangoni effect plays an important role in film evaporation. This study investigates the evaporation dynamics of ternary liquid thin films, revealing the occurrence of two different types of Bénard-Marangoni instabilities and elucidating their competing mechanisms. Our findings provide deeper insight into the evaporation behavior of multicomponent liquid films, aiding in the prediction and control of film morphology post-evaporation. Such knowledge is crucial for optimizing the performance and reliability of advanced materials and processes, particularly in the precise formation of functional coatings.

Evaporation of multicomponent liquid films is a fundamental and critical aspect for a wide range of industrial and scientific fields, including printing technologies (1), two-dimensional material manufacturing (2), organic photovoltaic fabrication (3), and biomolecular engineering (4). In these contexts, the ability to control the uniformity of the entire liquid film and to precisely manipulate the nucleation and deposition of materials is highly desirable (5–8), as it is essential for optimizing the performance and the quality of the final products. However, the physicochemical hydrodynamics of evaporating multicomponent films is inherently complex, arising from the intricate interplay between fluid flow, surface stresses, and heat and mass transfer processes (9, 10).

In the case of evaporating multicomponent droplets on a partially wetting substrate, the presence of the triple contact line not only generates a capillary flow inside the droplets (11) but also gives rise to the nonuniform evaporative flux and the associated Marangoni flows due to the emergence of gradients in the interfacial temperature and the solutal concentration (12–14). Furthermore, complex flow regimes within the evaporating droplet can occur (15–18), as a consequence of competition between different physical processes, such as capillary flow, thermal Marangoni flow, solutal Marangoni flow, gravity-induced flow, etc. When the droplet is perfectly wetted at the surface and becomes a liquid film without contact lines, the internal capillary flow and the nonuniform evaporation effects are notably suppressed (12). This feature enables us to comprehensively investigate the evaporating dynamics of multicomponent thin films, and especially the convective instabilities involved in this process (19–23). The different properties of multiple components, such as surface tension and volatility, significantly influence these instability phenomena (24, 25).

Here, we focus on the evaporation dynamics of a liquid film of an ouzo mixture, consisting of ethanol, water, and trans-anethole oil. This mixture has been widely utilized in studying physicochemical hydrodynamic problems, due to its preferential evaporation characteristics and the phase separation features associated therewith (26–29). We observe the formation of convective cells during evaporation through thermal imaging and the optically observable patterns created by the local nucleation of oil droplets, wherever the ethanol concentration has become low enough. By regulating the timing of the droplet nucleation (through the initial ethanol concentration), convection patterns with different wavelengths are obtained, which clearly distinguish between convection caused by solutal and by thermal instability mechanisms. We then compare the strengths of these two effects based on the temperature and concentration fields obtained from the numerical simulations. Finally, the findings are used to control and modulate the deposition pattern of an evaporating colloidal liquid film to demonstrate the application perspectives of our findings.

## Results

### Patterns Formed During the Evaporation Process.

**Fig. 1.**
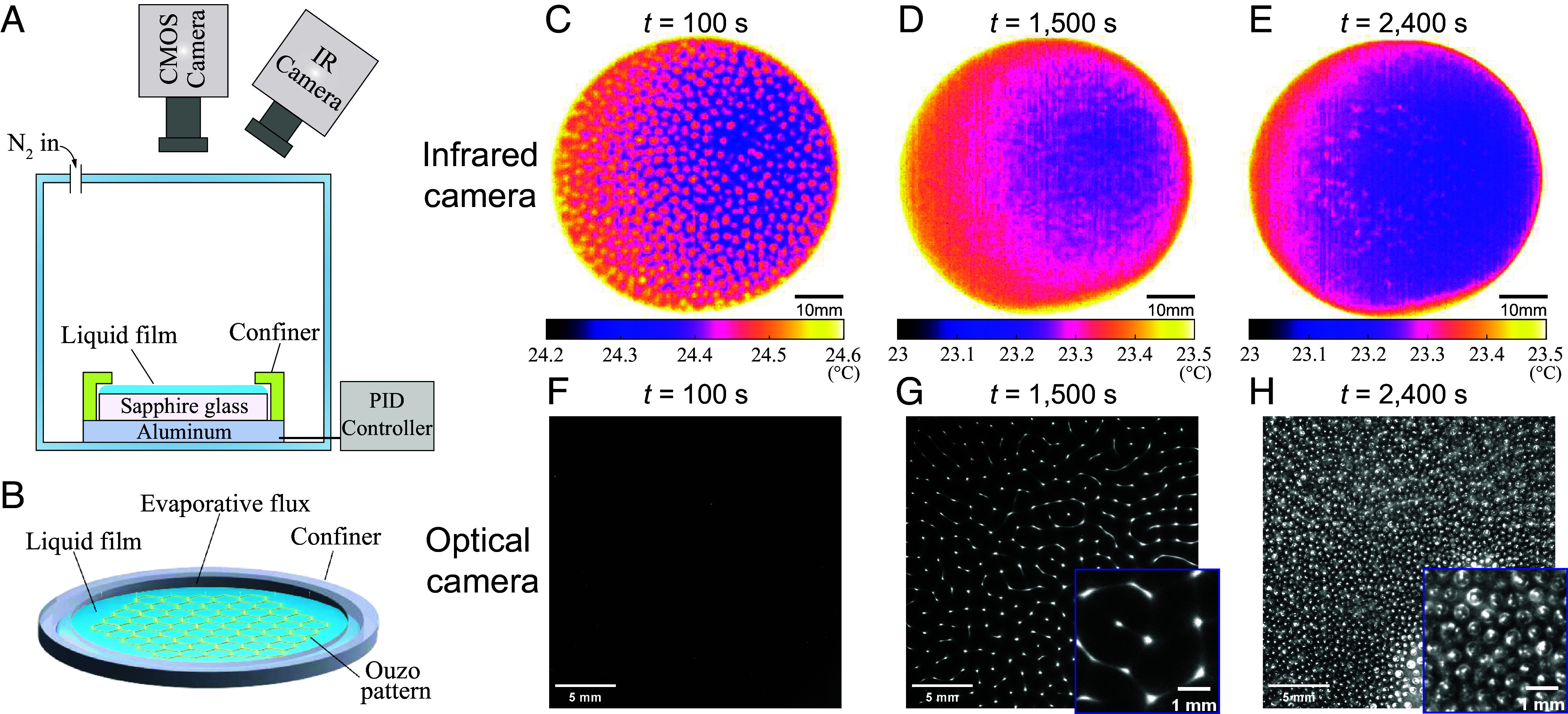
(*A*) Sketch of the experimental setup for observing the phenomena of liquid film evaporation. The liquid film completely covers the sapphire substrate (with 60 mm diameter), with a ring-shaped confiner (with 58 mm inner diameter) at the edge of the liquid film for limiting evaporation at the boundary contact line. Observations are conducted by an infrared camera and an optical CCD camera in the top view. (*B*) 3D schematic diagram showing the evaporation of liquid film in the presence of the confiner. The presence of the confiner eliminates the contact line effect and allows the evaporation in the center portion of the surface to be treated as that of a flat, infinite liquid film (see *SI Appendix* for more details). Time series images (t=100,1,500,2,400 s) obtained by the infrared camera (*C*–*E*) and optical camera (*F*–*H*) of the evaporating ouzo liquid film (at 10% RH, with initial film height h=0.55 mm). (*C* and *F*) Shortly after the deposition of the liquid film, a thermal pattern with small round specks is observed in the thermal map, while the liquid film remains transparent and no pattern is observed in the optical camera. (*D* and *G*) The thermal pattern gradually disappears and the temperature becomes uniform, while polygonal patterns caused by the precipitation of oil microdroplets can be observed in the optical camera. (*E* and *H*) Oil microdroplets aggregate to form larger droplets that spread over the surface of the liquid. The tiny spots seen on the infrared image correspond to small oil droplets that nucleate and aggregate. The temperature field on the surface of the liquid is homogeneous, corrected by emissivity calibration. Videos of the process obtained by optical and infrared cameras can be found in Movies S1 and S2, respectively.

The miscible and transparent ouzo solution, composed of 75% (vol/vol) ethanol, 15% (vol/vol) water, and 10% (vol/vol) trans-anethole oil (Case 1 in Table 1), is gently injected onto a sapphire substrate by a milliliter syringe at room temperature, forming a thin liquid layer with an aspect ratio h/d≈0.01 (thickness h≈0.55 mm and diameter d≈60 mm). The evaporation process is observed using an infrared camera and a CCD camera (Fig. 1*A*). To further weaken the effect of the contact line on the evaporation flux and the internal flow (30, 31), a ring-shaped confiner is positioned above the liquid film (Fig. 1*B*; see *Materials and Methods* for details).

**Table 1.** The conditions for the three cases in the experiments and the corresponding $t_{\mathrm{thermal}}$ and $t_{\mathrm{ouzo}}$

		Composition (vol/vol)		
Case	$T_{\text{sub}}{(}^{{}^{\circ}}\text{C})$	EtOH:Water:trans-Anethole	$t_{\text{thermal}}\left(\text{s}\right)$	$t_{\text{ouzo}}\left(\text{s}\right)$
1	25	75:15:10	500	1,200
2	25	72:16:12	400	250
3	15	75:15:10	/	400

**Fig. 2.**
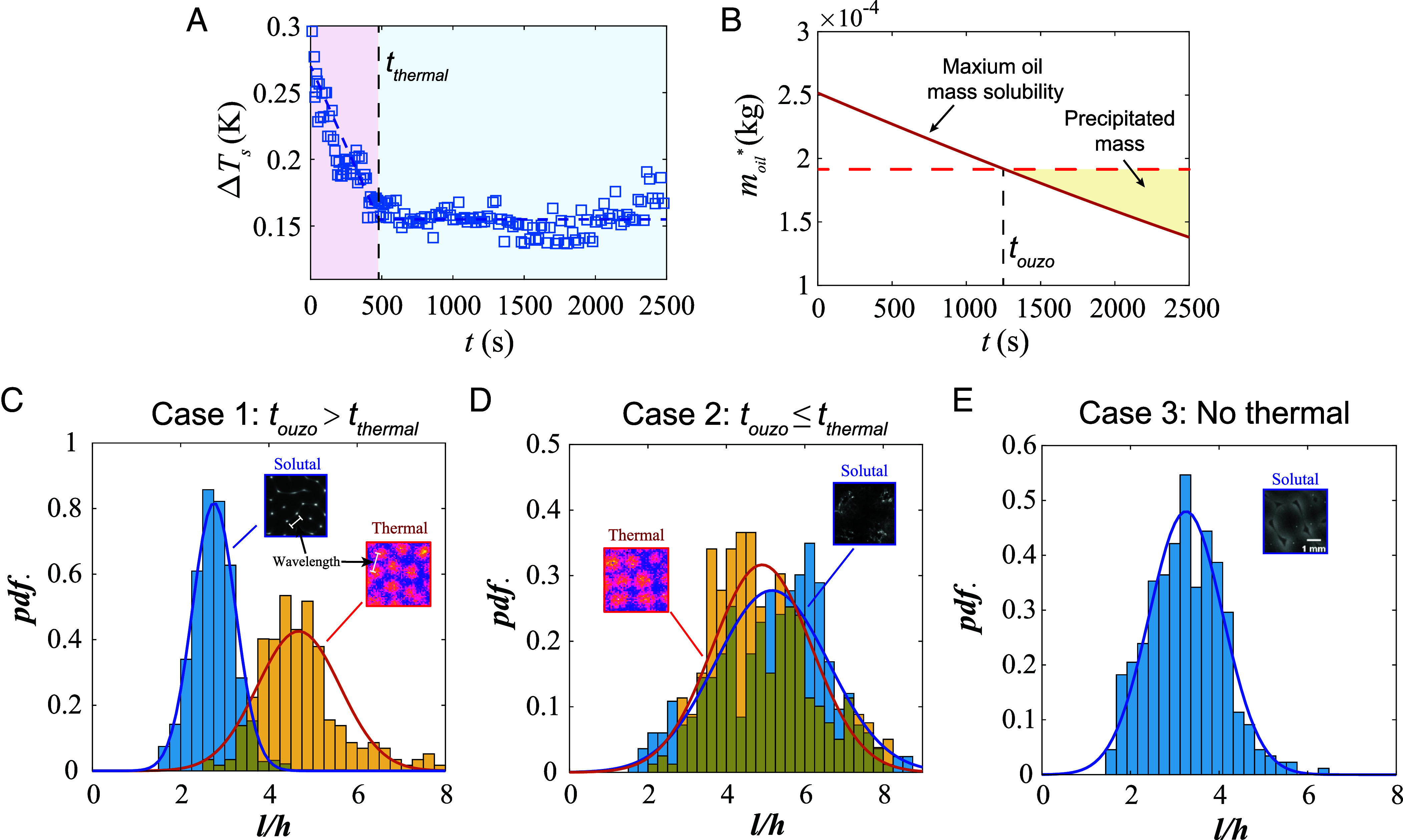
(*A*) Temporal variation of the temperature fluctuation $\Delta T_{s}$ of the upper liquid surface. The transition moment from the temperature fluctuation decline stage to the stabilized stage is defined as $t_{\text{thermal}}$. (*B*) Mass solubility of trans-anethole oil $m_{\mathrm{oil}}^{*}$ and the precipitated mass in the mixture over time. The decreasing maximum soluble oil (solid line) intersects with constant oil mass (dashed line) at $t_{\text{ouzo}}$, indicating the precipitation onset. The phase separation point is calculated based on the current mixture composition obtained by the numerical model. (*C*–*E*) Probability density functions (pdf) of the dimensionless wavelength l/h of the thermal pattern observed by the infrared camera and the ouzo pattern observed by the CCD camera. h is the typical liquid film thickness at the moment of the first appearance of the corresponding pattern. The results of three cases are shown, corresponding to different relationships of $t_{\text{thermal}}$ and $t_{\text{ouzo}}$. The experimental conditions are listed in Table 1. The *Insets* show the typical images of the patterns and the full process is shown in Movies S1–S4.

Immediately following the deposition of the liquid film, periodic cellular patterns are observed in the infrared thermal image (Fig. 1*C*), which are reminiscent of the hexagonal thermal Bénard-Marangoni convection cell when the thin fluid layer is heated from below (19, 20, 32, 33). In the current case, the temperature difference driving the convection is caused by the evaporative cooling effect (23, 34, 35). The generated cellular thermal pattern is macroscopically uniform in both size and distribution, which is different from previous observations in evaporating droplets (18, 36). The typical temperature difference within one convection cell $\Delta T_{s}$ decreases slowly from 0.3 K to 0.15 K, with a rate of $3\times 10^{-4}$ K/s, until a critical time (defined as $t_{\text{thermal}}$) after which $\Delta T_{s}$ remains almost constant (Fig. 2*A*).

In contrast to the infrared view, observations made using an optical camera reveal that the liquid film remained transparent for a period of time after the onset of the evaporation process, with no observable pattern (Fig. 1*F*). During the preferential evaporation of ethanol, the ethanol-concentration-dependent saturation concentration of trans-anethole oil decreases, gradually approaching and crossing the phase separation line at the transition time point $t_{\text{ouzo}}$ [as calculated in Fig. 2*B*, the evaporation model can be found in previous work by Wakata et al. (37)], resulting in oil microdroplets precipitating out and becoming visible in the optical camera (38, 39). Unlike the uniformly milky white ouzo emulsion observed in evaporating droplets (26, 27) which emerges due to the strong internal mixing flow, here the liquid film exhibits a regular polygonal pattern, as shown in Fig. 1*G*. As the oil microdroplets continue to precipitate and merge, larger oil droplets form by growth and coalescence and spread across the surface of the liquid (Fig. 1*H*). Throughout the patterning process induced by the droplet nucleation, the surface temperature measured by the infrared camera remains uniform (Fig. 1*D*) until the merging of oil droplets causes inhomogeneity in the surface thermal map (Fig. 1*E*).

### Wavelength Distribution of Different Cases.

To gain a more quantitative understanding of the observed patterns, we first define two typical timescales: $t_{\text{thermal}}$ is the time when the surface temperature field variation becomes constant, and $t_{\text{ouzo}}$ relates to the moment when the liquid mixture reaches the phase separation line (Fig. 2 *A* and *B*). In the current case (Case 1), $t_{\text{thermal}}\approx 500\;\;\text{s}$ and $t_{\text{ouzo}}\approx 1,200\;\;\text{s}$, which means that the microdroplet nucleation occurs long after the disappearance of the thermal pattern, indicating two different mechanisms for the occurrence of these two patterns. We analyze the mean wavelengths of the pattern captured by the infrared camera (thermal pattern) and by the optical camera (oil droplet pattern), calculated as the mean distance between two adjacent bright spots in the infrared or optical images (see the *Inset* of Fig. 2*C*). The probability density function (pdf) of the dimensionless wavelength (l/h) for the aforementioned case (Case 1 in Table 1, $t_{\text{ouzo}}>t_{\text{thermal}}$) is illustrated in Fig. 2*C*. It can be seen that the wavelength of the ouzo pattern (l/h≈3) is approximately twice as small as that of the thermal pattern (l/h≈5). The effect of film thickness reduction during evaporation can be excluded as a primary factor, as it accounts for only approximately 10% of the total dimensionless wavelength reduction, ruling out thickness dependence as the main cause of the observed wavelength discrepancy (23, 40, 41). It should be noted that the wavelengths of both patterns exhibit remarkable stability during their respective formation stages (*SI Appendix*, Fig. S3). These distinct yet individually constant wavelengths strongly indicate the presence of two different controlling mechanisms.

To further investigate the underlying cause of the discrepancy in the wavelength of the two patterns, two further cases were studied: one in which an ouzo solution closer to the phase separation line is used (Case 2, $t_{\text{ouzo}}<t_{\text{thermal}}$), and another in which the substrate temperature was decreased to remove the thermal pattern (Case 3).

We find that when the microdroplet nucleation occurs along with temperature fluctuation (Case 2), the wavelengths of the two patterns are similar, also consistent with the thermal pattern of Case 1 (Fig. 2*D*), while the wavelength in Case 3 (with no thermal pattern) exhibits a strong resemblance to that of the ouzo pattern in Case 1 (details in *SI Appendix*), but differs significantly from the results observed in Case 2.

**Fig. 3.**
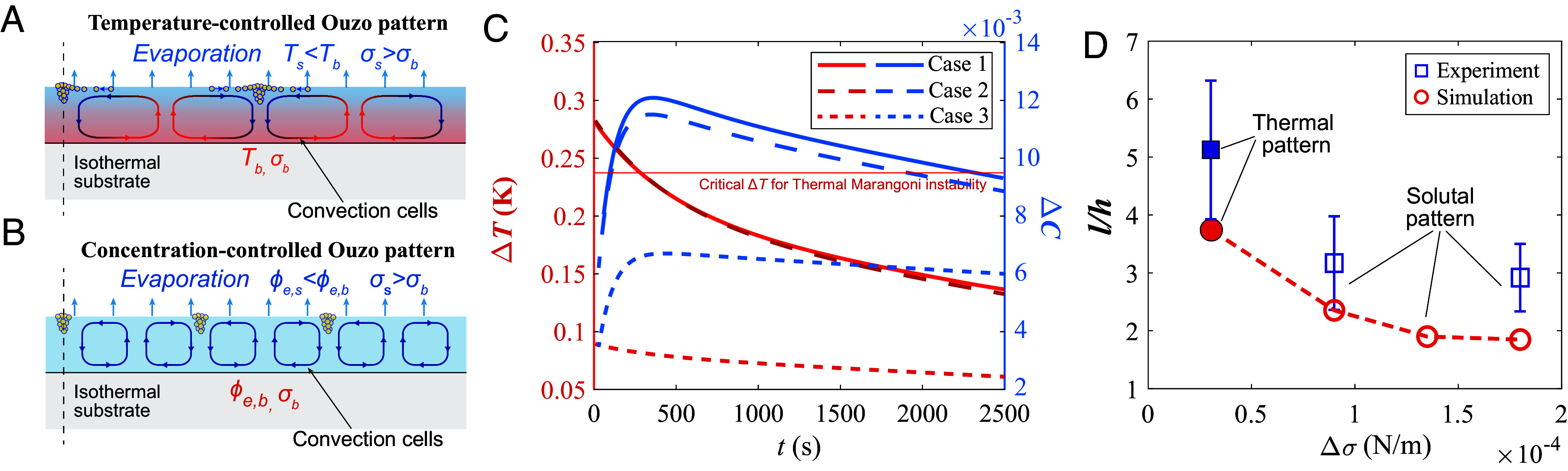
(*A*) Sketch of the formation of ouzo pattern due to thermal Bénard–Marangoni instability. Evaporation leads to a decrease in the surface temperature of the liquid film ($T_{s}<T_{b}$), which increases the surface tension ($\sigma_{s}>\sigma_{b}$) and causes the appearance of a convection cell. The subscripts s,b stand for surface and bottom, respectively. If $t_{\text{ouzo}}<t_{\text{thermal}}$, oil microdroplets will be precipitated at this stage following the motion of the convection cell and reflect the patterns on the surface. (*B*) Sketch of ouzo pattern due to solutal Bénard–Marangoni instability. The greater evaporation rate of ethanol leads to a lower ethanol concentration at the surface ($\phi_{e,s}<\phi_{e,b}$), which also causes a surface tension gradient ($\sigma_{s}>\sigma_{b}$). (*C*) Numerical results of temperature difference ΔT and ethanol concentration difference ΔC between the upper and bottom liquid surface. Numerical results of the three cases and the corresponding experimental conditions are listed in Table 1. (*D*) Dimensionless pattern wavelength l/h as a function of surface tension difference between the upper and lower surface of the liquid film Δσ. Results of experiments and simulations are shown. The dashed line shows the trend.

Given that the oil microdroplets produced by the ouzo effect can be regarded as tracer particles reflecting the local flow field (27), the differing wavelengths of the ouzo patterns in the three cases further confirm the existence of two controlling mechanisms in the formation of the patterns, as sketched in Fig. 3 *A* and *B*. During the evaporation process, a negative temperature gradient and a negative concentration gradient of ethanol in the vertical direction arise due to the evaporative cooling effect and the preferential evaporation of the more volatile ethanol component, respectively (37). Both factors lead to a higher surface tension. For a random increase in the local evaporation rate, the decreasing local ethanol concentration and the resulting decreasing local temperature (due to evaporative cooling) will both increase the local surface tension. The resulting surface tension difference drives the motion of the surrounding liquid and generates convective cells in the liquid bulk, manifesting as Bénard–Marangoni convection (24, 25). In Case 2 ($t_{\text{ouzo}}<t_{\text{thermal}}$), the oil droplet nucleation occurs when the temperature is inhomogeneous, and the small oil droplets move in accordance with the flow generated by the thermal Marangoni convection, thereby forming an ouzo pattern with a wavelength that is consistent with that of the thermal (Fig. 3*A*). Conversely, in Case 1 and Case 3, the oil droplet nucleation phenomenon occurs when there is no longer a temperature difference, and the ouzo pattern reflects the wavelength of the solutal Bénard–Marangoni convection (Fig. 3*B*).

### Numerical Analysis.

To elucidate the distinct contributions of thermal and solutal Marangoni effects in evaporating thin films, we conduct numerical calculations using the finite element framework based on OOMPH-LIB (42) and GiNaC (43) to obtain the flow field as well as the temperature and the concentration distribution. These frameworks properly consider both the thermodynamics and the hydrodynamics of the evaporating multicomponent liquid film. A comprehensive explanation of this finite element model can be found in refs. 44 and 45. More details on the numerical model are listed in *Materials and Methods*.

We first focus on the temperature and concentration difference between the upper and lower surface of the liquid layer (Fig. 3*C*), as it determines whether or not instabilities occur. The critical temperature and concentration differences driving the two Bénard–Marangoni instabilities can be obtained from linear stability analysis (20, 23, 46), which is around 0.24K for the critical temperature difference and smaller than $2\times 10^{-5}$ for the critical concentration difference, both under the current conditions of ouzo liquid film evaporation; see *SI Appendix* for the detailed calculations.

For all three cases in the numerical simulation, the temperature difference ΔT monotonically decreases with time. In contrast, the concentration difference ΔC exhibits a nonmonotonic variation with time, indicating a diffusion-limited process of mass transfer due to the large Lewis number $Le=\kappa_{l}/D_{l}\approx 100$, where $\kappa_{l}$ and $D_{l}$ are the thermal diffusivity and mass diffusivity of the liquid, respectively. Furthermore, it can be seen that the calculated temperature difference of Case 1 and 2 is near 0.3 K, which is larger than the critical temperature difference for the onset of convection and consistent with the experimental data in Fig. 2*A*, while the temperature difference of Case 3 is below the critical temperature, which explains why there is no thermal pattern in Case 3. On the other hand, the concentration differences of the three cases are all above the critical value, explaining why the solutal Marangoni instability will be induced.

We analyze the formation of convection cells by thermal and solutal effects by testing the cases where the surface tension was only affected by temperature and only by concentration, respectively. Convection rolls can be observed in the flow field (*SI Appendix*, Fig. S6 *A* and *B*). *SI Appendix*, Fig. S6*C* shows the distribution of the vertical velocity in the radial direction. The typical difference of surface tension Δσ can be decomposed as $\Delta \sigma =\Delta \sigma_{T}+\Delta \sigma_{S}$ (10, 47, 48), where $\Delta \sigma_{T}=\partial_{T}{\sigma |}_{C}\Delta T$ and $\Delta \sigma_{S}=\partial_{C}{\sigma |}_{T}\Delta C$ are the surface tension change corresponding to the temperature and concentration, respectively. Fig. 3*D* shows the relationship between Δσ and the dimensionless wavelength l/h. It can be seen that, independently of whether the Marangoni flow is triggered by gradients of temperature or concentration, the typical wavelength l/h decreases with the increase of Δσ (49), and the simulation results agree well with the experimental results. These results further confirm the existence and transition of dual Bénard-Marangoni instabilities within evaporating thin multicomponent liquid films.

### Transition of the Two Dominant Stages.

Based on the experimental observations and numerical simulations, we now turn to figure out the dominant phases of these two instabilities. Here, due to the large Lewis number of liquids, we need to consider the transient boundary layers for both the temperature (of thickness $\lambda_{T}$) and concentration (of thickness $\lambda_{C}$), as explained in ref. 46. $\lambda_{T}=\;\min\left(\sqrt{\kappa t},h\right)$ is the diffusion length scale of heat transport and $\lambda_{C}=\;\min\left(\sqrt{\mathrm{Dt}},h\right)$ is the diffusion length scale of mass transport. Then, the contributions of temperature and concentration on the surface tension difference can be compared as follows:

[1] $$\frac{\Delta \sigma_{T}}{\Delta \sigma_{S}}=\frac{\partial_{T}{\sigma |}_{C}\Delta T}{\partial_{C}{\sigma |}_{T}\Delta C}=\frac{\partial_{T}{\sigma |}_{C}\cdot \partial_{z}T\cdot \lambda_{T}}{\partial_{C}{\sigma |}_{T}\cdot \partial_{z}C\cdot \lambda_{C}},$$

where $\partial_{z}T$ and $\partial_{z}C$ are the typical gradients of temperature and concentration within the liquid film, which can be obtained by numerical simulations.

**Fig. 4.**
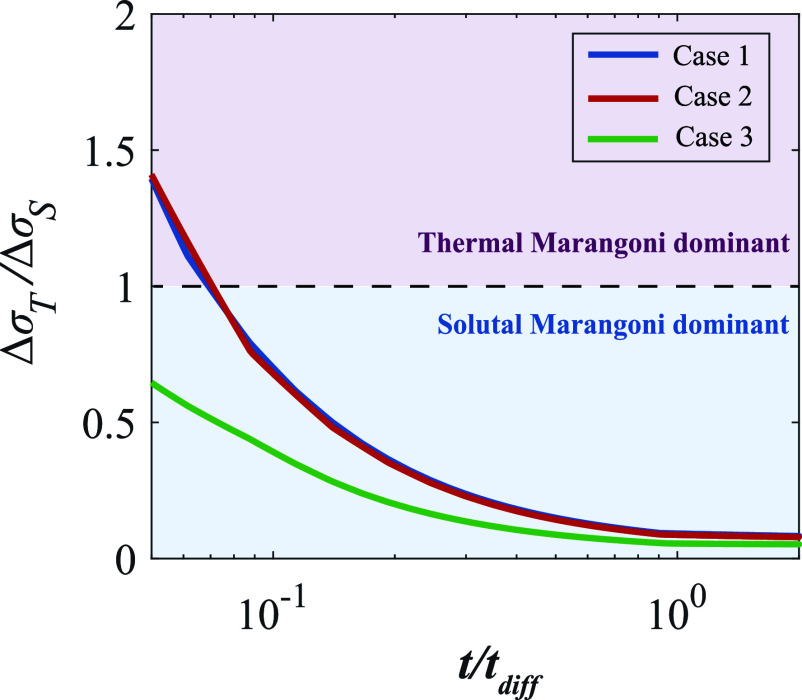
Ratio between the surface tension difference induced by temperature and concentration $\Delta \sigma_{T}/\Delta \sigma_{S}$ as a function of the nondimensional time $t/t_{\text{diff}}$. Here, $t_{\text{diff}}=h_{i}^{2}/D\approx 250\;s$ is the diffusion time where $h_{i}$ is the initial liquid height and D the diffusion coefficient of ethanol in the mixture. $\Delta \sigma_{T}/\Delta \sigma_{S}$ greater than 1 corresponds to the thermal Marangoni dominant stage and vice versa for the solutal Marangoni dominant stage. Numerical results of the three cases and the corresponding experimental conditions are listed in Table 1.

The temporal variation of $\Delta \sigma_{T}/\Delta \sigma_{S}$ calculated through the numerical results is shown in Fig. 4. It can be seen that for Cases 1 and 2, $\Delta \sigma_{T}/\Delta \sigma_{S}$ is found to be greater than 1 at the initial stage, indicating that the thermal instability is more pronounced at this period, and then the ratio $\Delta \sigma_{T}/\Delta \sigma_{S}$ decreases gradually with time, approaching 0, reflecting that the solutal Marangoni effect dominates at all subsequent times. This nonmonotonic evolution of $\Delta \sigma_{T}/\Delta \sigma_{S}$ arises from the competition between the thermal diffusion and the much slower mass diffusion through the liquid film, which enables the thermal Marangoni effect to be dominant at the early stage of evaporation. For Case 3, $\Delta \sigma_{T}/\Delta \sigma_{S}$ is found to be smaller than 1 during the entire process of evaporation, demonstrating that the thermal Marangoni effect is not pronounced, which is consistent with the absence of thermal patterns in experiments (Fig. 2*E*).

The phase diagram (Fig. 4) enables us to modulate the typical wavelength l/h of drying thin liquid films, by means of temperature and concentration. Long-wave cellular patterns can be produced by the thermal Marangoni effect, which requires not only the high substrate temperature $T_{\text{sub}}$ but also fast precipitation of oil droplets $t_{\text{ouzo}}<t_{\text{thermal}}$, corresponding to Case 2 (Fig. 2*D*). Short-wave cellular patterns can be produced by the solutal Marangoni effect, which is more robust than the thermal approach, corresponding to Case 1 and 3 (Fig. 2*C* and *E*).

**Fig. 5.**
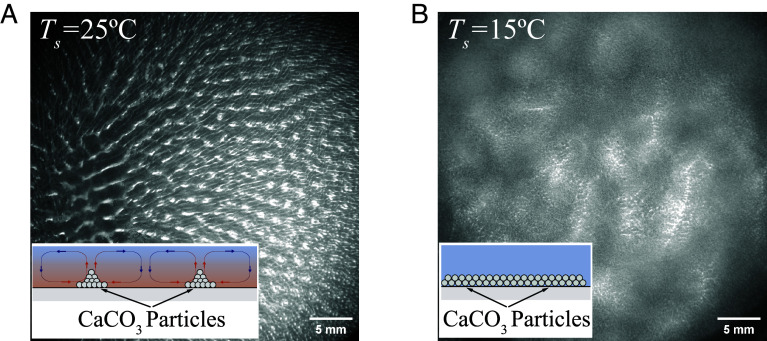
Final deposition pattern of a drying liquid layer of water-ethanol solution dispersion of calcium carbonate particles (${\mathrm{CaCO}}_{3}$) on substrates with temperature *T*_sub_ = 25 °C (*A*) and *T*_sub_ = 15 °C (*B*). *Insets* show the schematic representation of the mechanism of the particle deposition.

In Fig. 5, we investigate the final deposition pattern of a water-ethanol liquid film containing suspended particles of calcium carbonate (CaCO_3_, $r_{p}=1\mu m,\rho_{p}=2.7\times 10^{3}\;\;{\text{kg/m}}^{3}$) with an initial mass fraction $\phi_{p}=2$%. Due to a slightly higher density than the water-ethanol mixture, the particles will sediment slowly, with a deposition time scale $t_{\text{deposition}}=h/v_{t}\approx 260\;s$, where $v_{t}$ is the terminal velocity of particle descending calculated by Stoke’s law (50). Two different substrate temperatures are employed in our experiments to give further evidence of the validity of our understanding of what controls the convective motion. For the substrate temperature *T*_sub_ = 25 °C, a regular pattern made by particle deposition is observed shortly after the spreading of the film (Fig. 5*A*), which is induced by thermal Bénard-Marangoni convection and is similar to our previous results in Fig. 1*G* and other published works (49). However, for a substrate with a lower temperature *T*_sub_ = 15 °C, the particles settle evenly on the substrate (Fig. 5*B*). It is known from the phase diagram in Fig. 4 that the thermal Marangoni effect at this surface temperature is insufficient to drive the convection. Moreover, although the solutal effect becomes pronounced later, the resulting solutal-induced convection is inadequate to dislodge the particles from the solid surface. Consequently, the particles settle uniformly downward and adhere to the wall, forming a relatively even layer without distinct patterns on the substrate.

## Conclusion and Outlook

In conclusion, we have experimentally and numerically investigated the evaporation process of a thin liquid layer of an ouzo mixture. Two distinct convection patterns, driven by thermal and solutal Bénard-Marangoni instabilities are observed through thermal mapping using an infrared camera and the observation of the oil microdroplets induced by the ouzo effect using an optical camera. We provide a comprehensive explanation for the formation of the wavelengths and explain the successive appearance of the thermal and solutal instabilities through the ratio of controlling dimensionless parameters $\Delta \sigma_{T}/\Delta \sigma_{S}$. The reported thermal-solutal-induced bistability can be further manipulated by regulating the thermal properties of the substrate and the volatility of the liquid components, as discussed in *SI Appendix*. Our findings can be extrapolated to much larger scales, and consequently pave the way for modulating the final drying pattern of multicomponent colloidal films.

## Materials and Methods

### Experimental Setup and Procedures.

The schematic diagram of the experimental setup is presented in Fig. 1*A*. In brief, the liquid mixture is deposited through a syringe onto a cylindrical sapphire substrate with 30 mm in radius, followed by drying under a confiner to reduce the evaporation at the edge of the liquid film. The geometry and the effects of the confiner are discussed in *SI Appendix*. The liquids used in the experiments can spread themselves on the liquid substrate due to the very small contact angle and the initial thickness of the liquid film is 0.55mm. An infrared camera (Telops FAST L200) and a CCD camera (Ximea XiD) with a long-distance microscope (Navitar) are placed in the top view to record the evaporation process. The ouzo mixture is prepared with ultrapure water, ethanol (≥99.7%), and trans-anethole oil (Sigma-Aldrich; 99%). The ternary phase diagram and the phase separation line of the mixture, as well as the physical properties of the three components, are given in *SI Appendix*, Fig. S2 and Table S1. The mixture is initially in the single-phase regime and then enters the biphasic regime. The evaporation experiments are conducted in a closed chamber with nitrogen flow input to maintain the ambient humidity below 10%. This is because moisture can significantly affect flow through water vapor condensation (51–53).

### Numerical Model.

The numerical calculation is performed using the finite element framework based on OOMPH-LIB (42) and GiNaC (43). A comprehensive explanation of this finite element model can be found in refs. 44 and 45. The liquid–gas interface is assumed to be undeformable. The liquid layer consists of the ternary mixture ouzo, consisting of ethanol, water, and trans-anethole oil. The gas layer consists of air (the absorption of which in the liquid is neglected) and the vapors of water and ethanol. The physical properties of the liquids and gases are listed in *SI Appendix*. The gas phase height is set to be 10 mm, which is 20 times larger than the liquid height. The lower surface of the liquid film region is defined as a constant temperature wall, and the upper surface of the gas phase region has a constant temperature at room temperature (*T* = 25 °C). The concentration of components at the gas phase boundary is arbitrarily given to match the experimental evaporation rate. To elucidate the individual effects of thermal and solutal Marangoni effects on the convection flow, we decouple the influences of the temperature and concentration on the surface tension, which allows us to examine the separate contributions of each factor to the convection rolls. The simulation is run until the phase separation stage of the ouzo mixture. The velocity fields of the thermal and solutal Marangoni effects are shown in *SI Appendix*, Fig. S6, enabling us to measure the wavelength of each case.

## Supplementary Material

Appendix 01 (PDF)

Movie S1. Optical imaging of the evaporation process of the ternary liquid film (Case 1: *T_sub_* = 25 °C, Ethanol: Water: Oil (vol/vol) =75:15:10). Polygonal patterns caused by the precipitation of oil micro-droplets can be observed at around 1400 s.

Movie S2. Infrared imaging of the evaporation process of the ternary liquid film (Case 1: *T_sub_* = 25 °C, Ethanol: Water: Oil (vol/vol) =75:15:10). Shortly after the deposition of the liquid film, a thermal pattern with small round specks can be observed in the thermal map. The pattern gradually disappears with time.

Movie S3. Optical imaging of the evaporation process of the ternary liquid film (Case 2: *T_sub_* = 25 °C, Ethanol: Water: Oil (vol/vol) =72:16:12).

Movie S4. Optical imaging of the evaporation process of the ternary liquid film (Case 3: *T_sub_* = 15 °C, Ethanol: Water: Oil (vol/vol) =75:15:10).

Movie S5. Evaporation process of water-ethanol solution dispersion of CaCO3 particles (*T_sub_* = 25 °C, Ethanol: Water (vol/vol) =75:25, Particle initial mass fraction *ϕ*p = 2%).

## Data Availability

All study data are included in the article and/or supporting information.
