# Supplementary material for: Thermal-solutal-induced bistability of evaporating multicomponent liquid thin films
Source: Proc Natl Acad Sci U S A. 2025 Feb 7;122(6):e2418487122. doi: 10.1073/pnas.2418487122 (PMC11831166; doi:10.1073/pnas.2418487122)
Supplement: Supplementary file 1 — Appendix 01 (PDF) [file pnas.2418487122.sapp.pdf]

Supporting Information for

Thermal-Solutal-Induced Bistability of Evaporating Multicomponent Liquid Thin Films

Yuki Wakata, Feng Wang, Chao Sun, and Detlef Lohse

Chao Sun
E-mail: chaosun@tsinghua.edu.cn

This PDF file includes:

Supporting text
Figs. S1 to S8
Table S1
Legends for Movies S1 to S5
SI References

Other supporting materials for this manuscript include the following:

Movies S1 to S5

Supporting Information Text

Section A: Effect of the confiner on the evaporation phenomenon

In this study, we utilized a ring-shaped confiner to control the effects of the contact line on the evaporation rate, ensuring that the central region of the liquid film remains unaffected by the flow due to the contact line effect. Figure S1A illustrates the scenario without a confiner, where the evaporation rate of ethanol is noticeably faster at the contact line compared with the center surface region. This increased rate leads to a higher surface tension in our ternary mixture of water, ethanol, and trans-anethole oil, which draws liquid at the interface towards the contact line. The decreased ethanol concentration leads to the nucleation of oil droplets, as evidenced by the pattern near the contact line shown in Fig. S1B. This phenomenon of phase segregation in evaporating droplets has been thoroughly studied in previous work (1, 2). However, unlike previous studies that focused on smaller droplets, our experiment examines a larger liquid film. As a result, we observed hexagonal patterns in the central region induced by the Bénard-Marangoni instability, similar to those described in our main text, even without a confiner. This observation inspired us to explore methods to weaken the influence of the contact line effect, aiming to enlarge the Bénard-Marangoni region in the center. Consequently, we designed the confiner system.

The confiner primarily consists of a ring-shaped plate positioned above the liquid film without direct contact, covering the boundary line area (see Fig. S1H and Fig. S1I). When a confiner with an appropriate ring diameter is used (which is also the case in the main manuscript), the evaporation rate becomes more uniform across the film, preventing additional flow due to the contact line effect. This scenario more closely resembles the evaporation process of an infinitely large liquid film. As a result, we can observe the stable and distinct Bénard-Marangoni patterns during the evaporation process, as evidenced in Fig. S1D. However, if the inner diameter of the confiner is too small, it results in the formation of an additional Marangoni ring (3), as illustrated in Fig. S1E. This occurs because the confiner reduces the evaporation rate in the area directly beneath it, leading to a lower surface tension in this region compared to the uncovered area. Consequently, this surface tension gradient induces a flow towards the center of the liquid film (see Fig. S1E). The visible ring formation in Fig. S1F clearly indicates this intensified flow towards the center. Additionally, employing a square-shaped confiner results in a square pattern (see Fig. S1G). This figure highlights the influence of the confiner's shape on the evaporation pattern, with the square geometry leading to distinct flow dynamics.

Section B: Phase diagram of the ouzo mixture system and the physical properties of the liquids

The ternary diagram of the ouzo system, consisting of water, ethanol, and trans-anethole oil, is plotted in Fig. S2. The two solid lines represents the measured phase-separation curve at 25 °C and at 15 °C reported in our previous work (4). The red dot indicates the initial concentration for Case 1, and the red arrow depicts the trajectory of the solution composition during evaporation. This trajectory crosses the phase separation boundary, entering the bi-phasic region as ethanol preferentially evaporates. The smaller red dots within the inset correspond to the simulated compositions at $t = 1000$ s and $t = 2000$ s, demonstrating the time-dependent progression of the system towards phase separation.

Table S1 shows the physical properties of the substances used in the experiment. Due to the unavailability of physical property data for ouzo mixtures, and the relatively low concentration of trans-anethole in the mixture ($< 15\%$), the viscosity, surface tension, and thermal conductivity of the mixture were estimated using data for binary water-ethanol solutions. The activity coefficients during the evaporation process were obtained using the UNIFAC model.

Section C: Critical Marangoni number for the onset of the Bénard–Marangoni instability

Here we focus on identifying the critical temperature and concentration differences that trigger the Bénard-Marangoni instability in drying liquid films. We analyze the critical parameters for Bénard-Marangoni instability due to the solutal Marangoni effect and the thermal Marangoni effect separately. The critical Marangoni number is adapted from Pearson's foundational work (5) with adjustments for the specific dynamics of evaporating films. Pearson's analysis used a small perturbation approach on an infinite, homogeneous liquid layer with a free upper surface, highlighting the surface tension variations due to temperature gradients and neglecting buoyancy forces. By linearizing the equations of motion and heat conduction and imposing boundary conditions at the liquid layer's surfaces, Pearson obtained a system of ordinary differential equations. The analysis provided critical values of the Marangoni number and corresponding wavenumbers, marking the onset of convective instability driven by surface tension.

Based on Pearson's framework, we adapted the Marangoni number formula to include evaporation effects from thermal and solutal scenarios (6, 7). The formula for the Marangoni number $Ma(k)$ is expressed as (5):

$$Ma(k) = \frac{8k(k\cosh[k] + \alpha k\sinh[k])(k - \sinh[k]\cosh[k])}{k^3\cosh[k] - \sinh^3[k]}, \quad \text{[S1]}$$

where $Ma(k)$ represents the Marangoni number as a function of the wavenumber k, α is a parameter that accounts for evaporation effects.

When analyzing the instability induced by the thermal Marangoni flow, we assume that the substrate temperature is fixed, which is consistent with our experiments. In Eq. S1, the parameter that accounts for evaporation effects α_T is given by:

$$\alpha_T = \frac{\lambda_g}{\lambda_l} + \frac{\mathcal{L}D_g M_{v,e} p'_{sat}(T_i^0)}{\lambda_l R T_i^0 (1 - y_i^0)}, \quad \text{[S2]}$$

where, λ_g and λ_l are the thermal conductivities of the gas and liquid phases, respectively. $\mathcal{L}$ is the latent heat of vaporization, D_g the diffusion coefficient of ethanol vapor in air, $M_{v,e}$ is the molar mass of the ethanol component, R is the universal gas constant, T_i^0 is the surface temperature in the unperturbed state, $p'_{sat}(T_i^0)$ is the derivative of the saturation pressure with respect to temperature, evaluated at T_i^0, y_i^0 is the mass fraction of vapor at the interface in the unperturbed state. We focus on ethanol evaporation effects on liquid film instability, as ethanol comprises about 70% of the liquid mass, far exceeding water's 15%. The steady-state liquid surface temperature T_i^0 is obtained through numerical simulation, ensuring accurate representation of the temperature distribution and its influence on the Marangoni instability.

For the solutal Marangoni effect, Eq. S1 can still be used by modifying the expression for α. We adopt the formula from (7) and consider relatively large gas-liquid thickness ratios:

$$\alpha_S = \frac{\rho_g D_g}{\rho_l D_l} \frac{(1-c_b)}{\left[1 + c_b\left(\frac{M_{v,e}}{M_{v,w}} - 1\right)\right]^2} \frac{\gamma_e P_{sat,e}}{\frac{M_{v,a}}{M_{v,e}} P_0}, \quad \text{[S3]}$$

where, $\rho, D, M_v, \gamma, P_{sat}, P_0$ are the density, the diffusion coefficient, the molecular mass, the activity coefficient, the saturation vapor pressure, and the room pressure, respectively. The subscripts w, e, a correspond to water, ethanol, and air respectively. c_b is the ethanol mass fraction at the bottom surface, which is assumed to be the same as the initial mass fraction.

Figure S5 presents the marginal curves of thermal and solutal Marangoni numbers against the wavenumber. The minimum Marangoni number relates to the critical value that triggers the Bénard-Marangoni instability. We can calculate the critical temperature difference and concentration difference by using the formula:

$$Ma_T = \frac{\partial_T \sigma|_C \Delta T h}{\mu_l \kappa_l}, \quad \text{[S4]}$$

$$Ma_S = \frac{\partial_C \sigma|_T \Delta C h}{\mu_l D_l}, \quad \text{[S5]}$$

where $\sigma, \mu_l, \kappa_l, D_l$ are the surface tension, the viscosity, the thermal diffusivity, and the mass diffusivity of the liquids, respectively. h is the typical length scale.

In our experiments, the liquid film thickness $h = 0.55$ mm, $\partial_T\sigma|_C = -1 \times 10^{-4}$ N/(m · K), $\partial_C\sigma|_T = -0.0218$ N/m . Thus, the corresponding critical temperature difference $\Delta T = -0.24$ K and critical ethanol mass concentration difference $\Delta C = -1.4 \times 10^{-5}$.

Section D: The effect of substrate temperature

The substrate temperature will affect the wavelength of the forming solutal pattern. As compared in Fig. S4, the PDF of the dimensionless wavelength of the solutal pattern in Fig. 2C ($T = 25$ °C) is slightly narrower than that in Fig. 2E ($T = 15$ °C). Also, the mean value of the wavelength of the case in Fig.2C is slightly smaller. Regarding the differences in the PDFs of these two cases, we attribute them to the difference in substrate temperature. On the one hand, the decrease in temperature (Case 3, Fig. 2E, $T = 15$ °C) leads to a reduction in evaporation rate, which in turn decreases the concentration gradient and weakens the solutal Marangoni flow, as shown in Fig. 3C in the main text. As a result, the average wavelength in Case 3 is slightly larger corresponding to the smaller concentration gradient (surface tension gradient), as shown in Fig. 3D in the main text. On the other hand, the decrease in temperature leads to the decrease in the solubility of oil in Ouzo mixture, and therefore the oil pattern occurs earlier compared to Case 1 (Fig. 2C, $T = 25$ °C), potentially preventing the flow structure from fully developing, which may result in a slightly wider PDF.

Section E: The effect of thermal properties of the substrate

The substrate we used in the main manuscript is sapphire which has a high thermal conductivity. We also explored the use of a quartz substrate which has a lower thermal conductivity. We observed a pronounced ouzo pattern, with a wavelength consistent with that of the sapphire substrate. However, we faced challenges in capturing thermal patterns using infrared imaging on the quartz surface, probably due to a higher emissivity of quartz surfaces. This problem hindered direct comparisons.

Nevertheless, our numerical simulations indicate that the quartz substrate reduces the temperature difference between the upper and the lower surface of the liquid film (see Fig. S7). Specifically, the heat transfer from the lower surface of the liquid film to the upper surface cannot be compensated by heat transfer from the substrate, leading to a smaller temperature difference within the liquid film on the quartz surface. This diminished temperature gradient is expected to weaken thermal Marangoni effects and shorten the thermal regime, as shown in Fig. 3C in the main text.

Section F: The effect of volatility of the liquid components

Our numerical simulations suggest that when the volatility of the more volatile component increases (by increasing the vapor pressure of ethanol), the value of $\Delta\sigma_T/\Delta\sigma_S$ decreases, i.e., the solutal effect is more pronounced, as shown in Fig. S8.

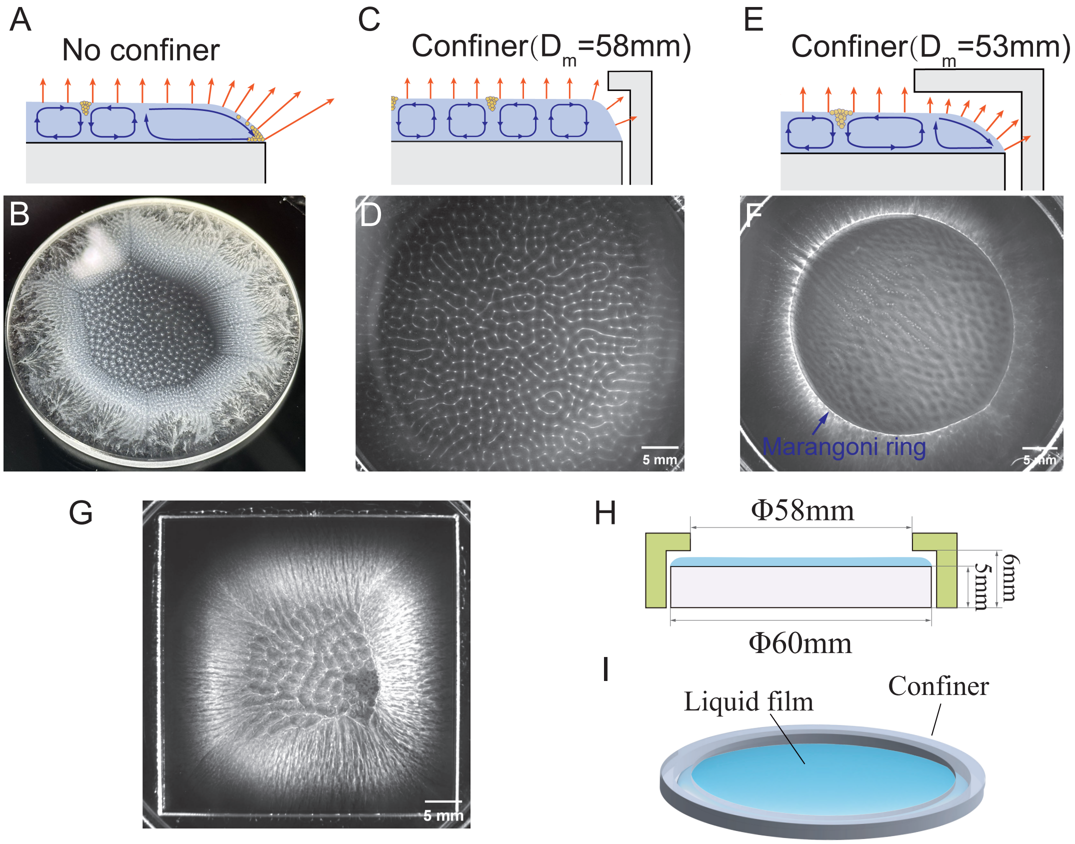

Fig. S1. Evaporation patterns with and without confiners of ouzo mixture. (A) Schematic of evaporation without a confiner, showing faster evaporation at the contact line. (B) Experimental result without a confiner, displaying oil droplet formation near the edge and Bénard-Marangoni (BM) patterns in the center. (C) Schematic of evaporation with a confiner with appropriate inner diameter $D_m = 58\,\mathrm{mm}$. (D) Experimental result with a confiner with an appropriate inner diameter, showing stable and distinct BM patterns across the surface. (E) Schematic of evaporation with a confiner with small inner diameter $D_m = 53\,\mathrm{mm}$, indicating flow towards the center. (F) Experimental result with the smaller confiner, demonstrating the formation of an additional Marangoni ring. (G) Evaporation pattern with a square-shaped confiner, highlighting the influence of confiner geometry. (H) Detailed geometry of the circular confiner used in the experiment in the main manuscript. (I) 3D schematic of the confiner and liquid film setup.

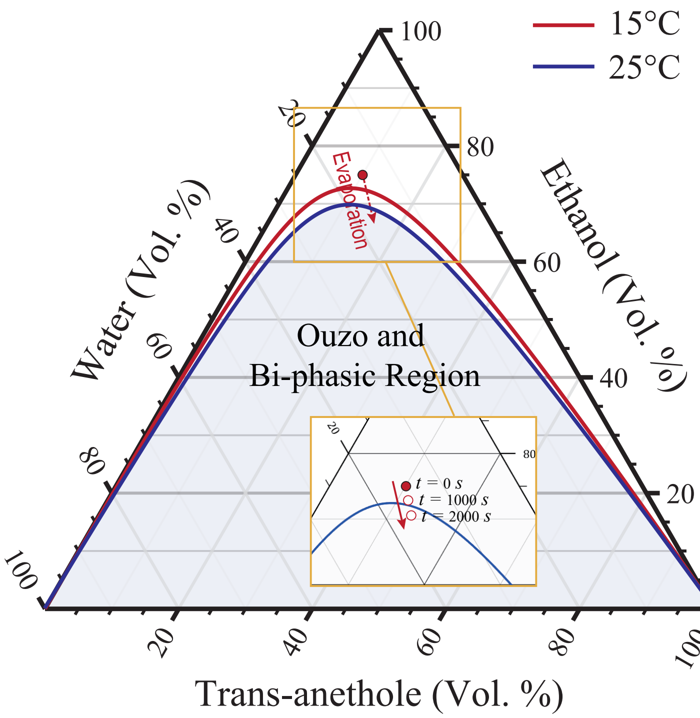

Fig. S2. Ternary diagram of the ouzo system, consisting of water, ethanol, and trans-anethole oil. The two solid lines represent the measured phase-separation curve at $25\,^{\circ}\mathrm{C}$ and at $15\,^{\circ}\mathrm{C}$ reported in our previous work (4). The red dot indicates the initial concentration for Case 1, and the mixture concentration at $t = 1000\,\mathrm{s}$ and $t = 2000\,\mathrm{s}$ (calculated by the numerical model) are labeled by the hollow points in the inset.

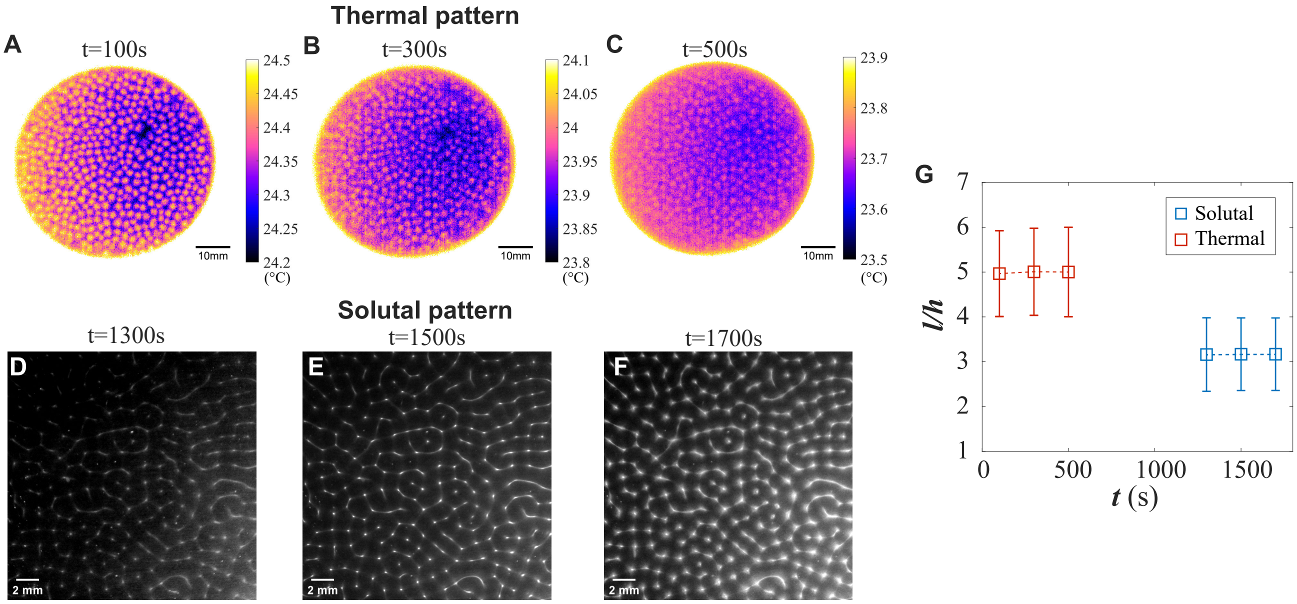

Fig. S3. (A-C) Time series images of the thermal pattern captured by infrared camera at $t = 100,\ 300,\ 500$ s. (D-F) Time series images of the solutal pattern captured by optical camera at $t = 1300,\ 1500,\ 1700$ s. (G) Non-dimensionalized wavelength l/h of thermal and solutal patterns as a function time. The red and blue symbols in the figure represent the wavelengths of thermal pattern and solutal pattern, respectively.

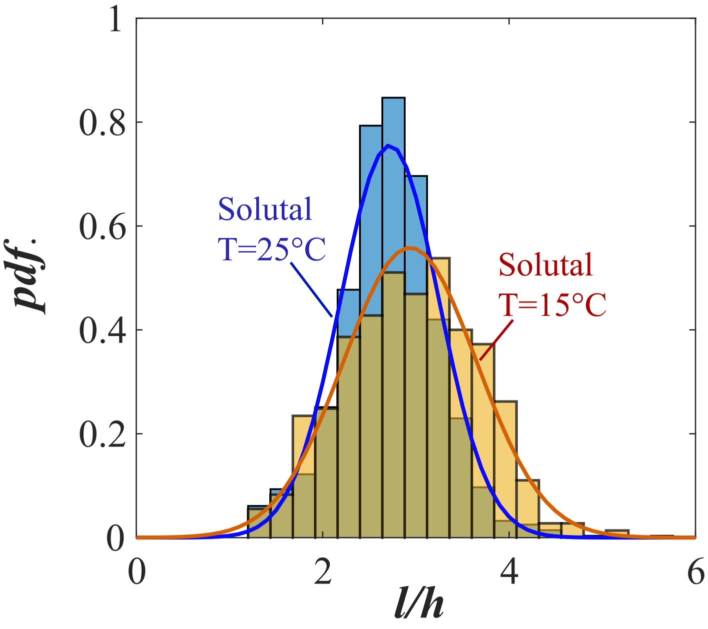

Fig. S4. Probability density functions (*pdf*) of the dimensionless wavelength l/h of the ouzo pattern at substrate temperature $T = 25\ ^{\circ}$C (Fig. 2C) and $T = 15\ ^{\circ}$C (Fig. 2E).

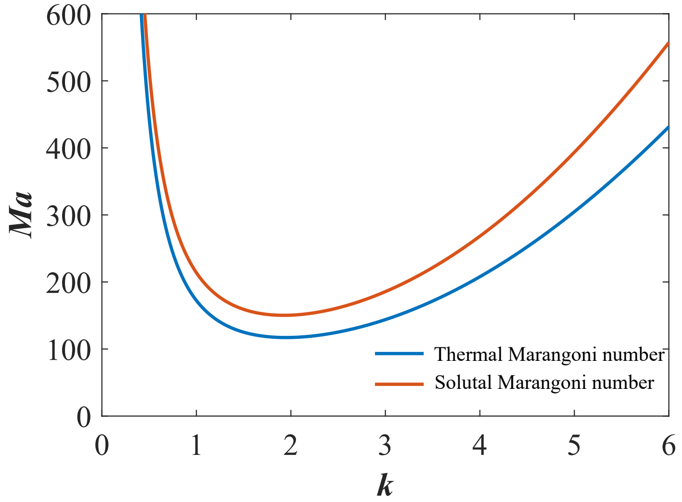

Fig. S5. The marginal curves of thermal and solutal Marangoni numbers against the wave number k. The minimum Marangoni number relates to the critical value that triggers the instability. The critical Ma of the thermal Marangoni instability is 112 and of the solutal Marangoni instability is 150.

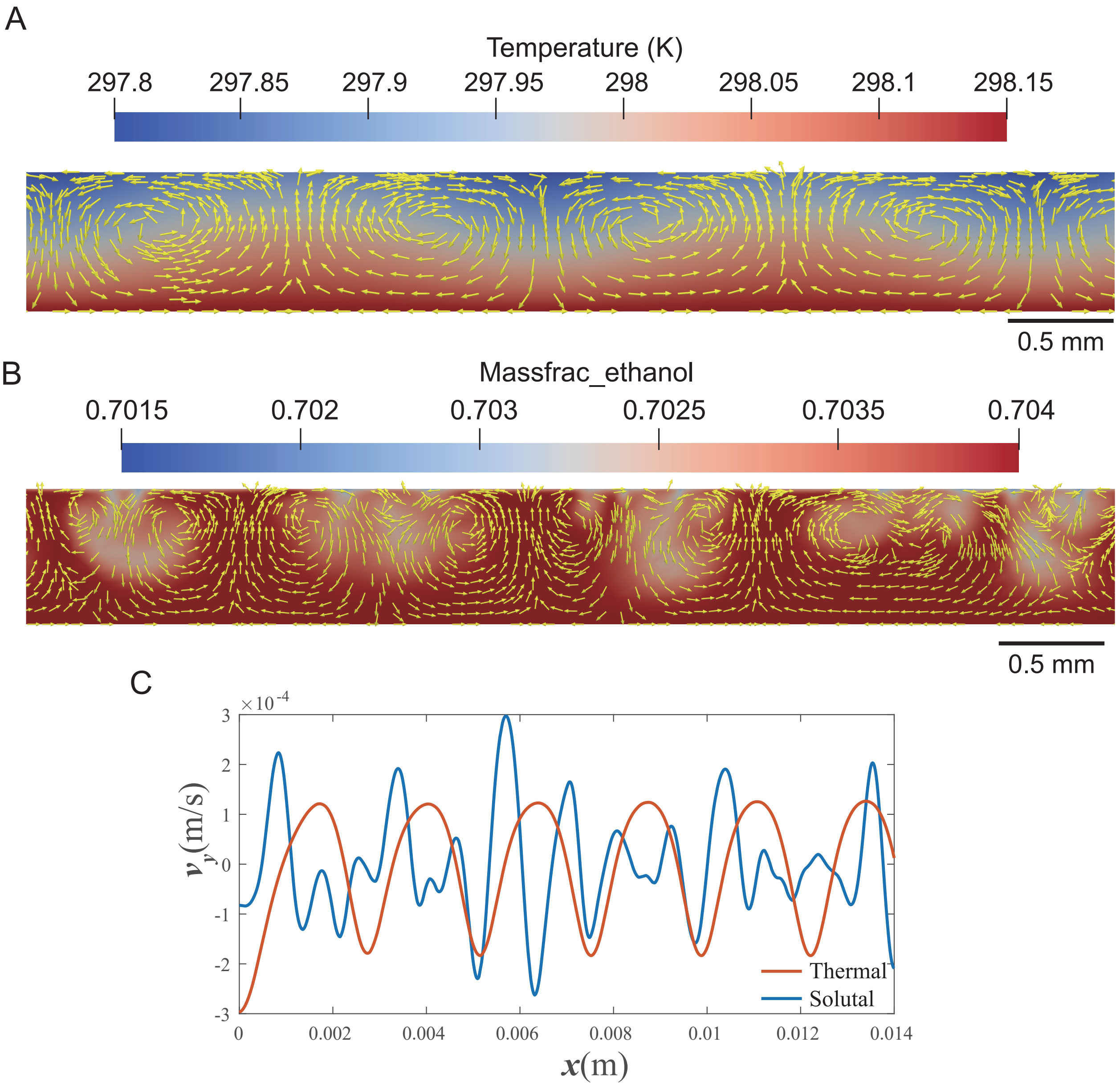

Fig. S6. (A) Simulated thermal-Marangoni driven Bénard–Marangoni instability. Temperature distribution when only the thermal Marangoni effect works at $t = 50\,\mathrm{s}$. The arrows represent the flow direction. (B) Simulated solutal-Marangoni driven Bénard–Marangoni instability. Mass fraction distribution when only the solutal Marangoni effect works at $t = 50\,\mathrm{s}$. The arrows represent the flow direction. (C) Distributions of vertical velocity v_y along the horizontal direction at the mid-height of the liquid film.

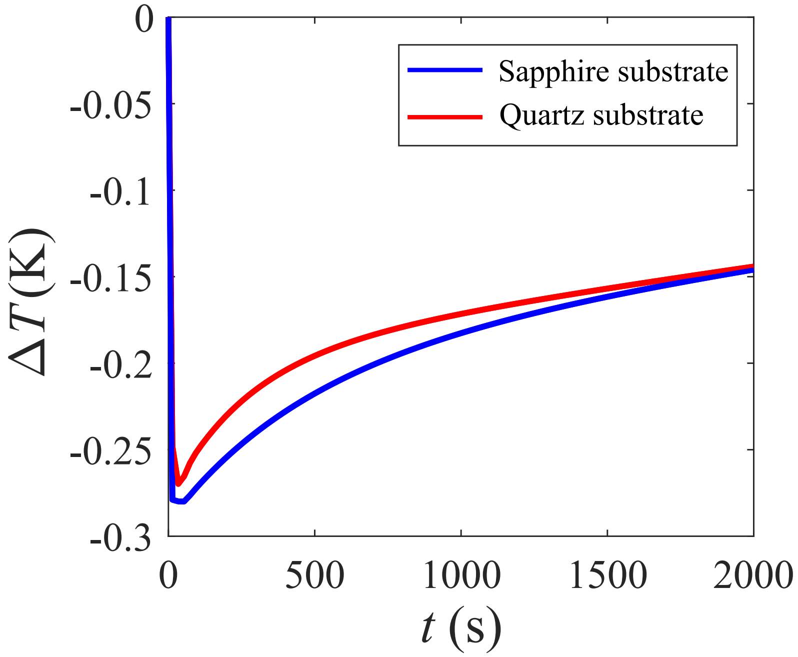

Fig. S7. Temporal variation of the surface temperature difference ΔT of the liquid film on sapphire substrate and quartz substrate.

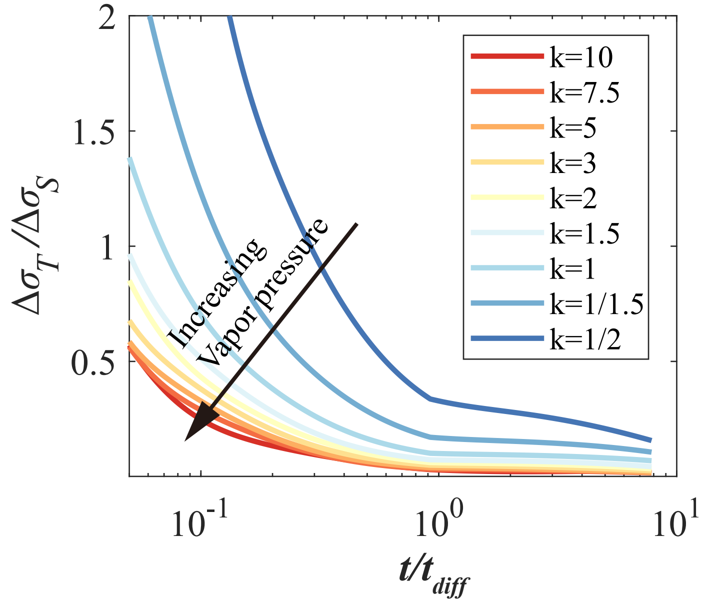

Fig. S8. Ratio between the surface tension difference induced by temperature and concentration $\Delta\sigma_T/\Delta\sigma_S$ as a function of the non-dimensional time t/t_{diff}.Here, $t_{diff} = h_i^2/D$ is the diffusion time, $k = P_{sat}/P_{sat,e}$ is the ratio of saturated vapour pressure of liquid to ethanol.

Table S1. Physical properties of the substances used in the experiment

Substance	Density ($\mathrm{kg/m^3}$)	Molar mass ($\mathrm{g/mol}$)	Thermal diffusivity ($\mathrm{m^2/s}$)	Viscosity ($\mathrm{Pa \cdot s}$)	Saturation vapor pressure (Pa)	Latent heat ($\mathrm{kJ/kg}$)
Water	997	18.015	0.145×10^{-6}	0.89×10^{-3}	3169	2256
Ethanol	785	46.07	0.082×10^{-6}	1.074×10^{-3}	7885	846
trans-Anethole	988	148.2	Not available	Not available	$133\,\mathrm{Pa}$ at $63\,^{\circ}\mathrm{C}$	Not available

Movie S1. Optical imaging of the evaporation process of the ternary liquid film (Case 1: $T_{sub} = 25\,°\mathrm{C}$, Ethanol: Water: Oil (vol/vol) =75:15:10). Polygonal patterns caused by the precipitation of oil micro-droplets can be observed at around $1400\,\mathrm{s}$.

Movie S2. Infrared imaging of the evaporation process of the ternary liquid film (Case 1: $T_{sub} = 25\,°\mathrm{C}$, Ethanol: Water: Oil (vol/vol) =75:15:10). Shortly after the deposition of the liquid film, a thermal pattern with small round specks can be observed in the thermal map. The pattern gradually disappears with time.

Movie S3. Optical imaging of the evaporation process of the ternary liquid film (Case 2: $T_{sub} = 25\,°\mathrm{C}$, Ethanol: Water: Oil (vol/vol) =72:16:12).

Movie S4. Optical imaging of the evaporation process of the ternary liquid film (Case 3: $T_{sub} = 15\,°\mathrm{C}$, Ethanol: Water: Oil (vol/vol) =75:15:10).

Movie S5. Evaporation process of water-ethanol solution dispersion of $CaCO_3$ particles ($T_{sub} = 25\,°\mathrm{C}$, Ethanol: Water (vol/vol) =75:25, Particle initial mass fraction $\phi_p = 2\%$).

References

1. H Tan, et al., Evaporation-triggered microdroplet nucleation and the four life phases of an evaporating Ouzo drop. *Proc. Natl. Acad. Sci. U.S.A.* **113**, 8642–8647 (2016).
2. Y Li, P Lv, C Diddens, D Lohse, Physiochemical hydrodynamics of the phase segregation in an evaporating binary microdroplet. *J. Fluid Mech.* **946**, A37 (2022).
3. L Thayyil Raju, et al., Evaporation of a Sessile Colloidal Water–Glycerol Droplet: Marangoni Ring Formation. *Langmuir* **38**, 12082–12094 (2022).
4. M Li, Y Wakata, H Zeng, C Sun, On the thermal response of multiscale nanodomains formed in trans-anethol/ethanol/water surfactant-free microemulsion. *J. Colloid Interface Sci.* **652**, 1944–1953 (2023).
5. JRA Pearson, On convection cells induced by surface tension. *J. Fluid Mech.* **4**, 489–500 (1958).
6. F Chauvet, S Dehaeck, P Colinet, Threshold of Bénard-Marangoni instability in drying liquid films. *Eur. Lett.* **99**, 34001 (2012).
7. H Machrafi, A Rednikov, P Colinet, Pc Dauby, Bénard instabilities in a binary-liquid layer evaporating into an inert gas. *J. Colloid Interface Sci.* **349**, 331–353 (2010).
